# Isolated high tibial osteotomy is appropriate in less than two-thirds of varus knees if excessive overcorrection of the medial proximal tibial angle should be avoided

**DOI:** 10.1007/s00167-020-06166-3

**Published:** 2020-07-20

**Authors:** Matthias J. Feucht, Philipp W. Winkler, Julian Mehl, Gerrit Bode, Philipp Forkel, Andreas B. Imhoff, Patricia M. Lutz

**Affiliations:** 1 Department for Orthopedic Sports Medicine, Technical University Munich, Ismaninger Str. 22, 81675 Munich, Germany; 2 Department of Orthopedics and Trauma Surgery, Medical Center, Faculty of Medicine, Albert-Ludwigs-University of Freiburg, Freiburg, Germany

**Keywords:** Varus, Osteotomy, HTO, Malalignment, Alignment

## Abstract

**Purpose:**

To perform a detailed deformity analysis of patients with varus alignment and to define the ideal osteotomy level (tibial vs. femoral vs. double level) to avoid an oblique joint line.

**Methods:**

A total of 303 digital full-leg standing radiographs of patients aged 18–60 years and varus alignment [mechanical tibiofemoral varus angle (mFTA) ≥ 3°] were included. All legs were analyzed regarding mFTA, mechanical medial proximal tibia angle (mMPTA), mechanical lateral distal femur angle (mLDFA), and joint line convergence angle. Based on mFTA, varus alignment was categorized as “mild” (3°–5°), “moderate” (6°–8°), or “severe” (≥ 9°). Deformity location was determined according to the malalignment test described by Paley. Two osteotomy simulations were performed with different upper limits for mMPTA: anatomic correction (mMPTA ≤ 90°, mLDFA ≥ 85°) and overcorrection (mMPTA ≤ 95°, mLDFA ≥ 85°). If a single osteotomy exceeded these limits at the intended mFTA of 2° valgus, a double-level osteotomy was simulated. If even a double-level osteotomy resulted in deviations from the defined limits, the leg was categorized as “uncorrectable”.

**Results:**

Mean mFTA was 6° ± 11° of varus (range 3°–15°). A tibial deformity was observed in 28%, a femoral deformity in 23%, a combined tibial and femoral deformity in 4%, and no bony deformity in 45%. The prevalence of a tibial deformity did not differ between varus severity groups, whereas a femoral and bifocal deformity was significantly more prevalent in knees with more distinct varus (*p* < 0.001). Osteotomy simulation revealed that isolated high tibial osteotomy (HTO) was appropriate in only 12% for anatomic correction, whereas a double-level osteotomy was necessary in 63%. If overcorrection of mMPTA was tolerated, the number of HTOs significantly increased to 57% (*p* < 0.001), whereas the number of double-level osteotomies significantly decreased to 33% (*p* < 0.001). Isolated DFO was considered ideal in 8% for both simulations. Significantly more knees were considered “uncorrectable” by simulating anatomic correction (18 vs. 2%; *p* < 0.001). A double-level osteotomy was significantly more often necessary in knees with “severe” varus (*p* < 0.001).

**Conclusion:**

Less than one-third of patients (28%) with mechanical varus ≥ 3° have a tibial deformity. If anatomic correction (mMPTA ≤ 90°) is intended, only 12% of patients can be corrected via isolated HTO, whereas 63% of patients require a double-level osteotomy. If slight overcorrection is accepted (mMPTA ≤ 95°), 57% of patients can be corrected via isolated HTO, whereas 33% of patients would still require a double-level osteotomy.

**Level of evidence:**

III, cross-sectional study.

## Introduction

Varus malalignment has historically been considered a tibial-based deformity and the broad majority of varus deformities are corrected via high tibial osteotomy (HTO) [3, 10, 19, 26]. However, recent studies have found highly variable coronal alignment in both, osteoarthritic and non-osteoarthritic knees [21, 23, 25, 34]. Based on these studies, varus malalignment can be the result of a tibial deformity, a femoral deformity, or a combined femoral and tibial deformity. Furthermore, varus malalignment may occur due to intraarticular wear and/or lateral ligament laxity without the presence of a bony deformity. Following the basic principles described by Dror Paley [39], osteotomies should be performed at the location of the deformity. If this rule is ignored, corrective osteotomies can result in an oblique joint line, which has been shown to negatively affect functional outcomes and survival after HTO [1, 4, 11, 47]. More specifically, if valgus HTO is performed in a normally aligned tibia, overcorrection of the mechanical medial proximal tibial angle (mMPTA) results in pathologic lateral inclination of the joint line [5, 39]. Excessive overcorrection of the mMPTA should be avoided, since studies have shown that a postoperative mMPTA of greater 95° leads to increased shear stress in the medial compartment and inferior clinical outcome [1, 37, 47]. Therefore, a femoral osteotomy [16, 51] or a combined tibial and femoral osteotomy (double-level osteotomy [5, 36, 41, 42, 45]) may be necessary in several patients to avoid an oblique joint line.

In a previous study, Eberbach et al. [13] have analyzed the geometry of valgus knees and found that a tibial deformity was most common, followed by a combined femoral and tibial deformity. Similar studies for varus knees are lacking. The purpose of this study was to perform a detailed deformity analysis of patients with varus malalignment and to define the ideal osteotomy level to avoid an oblique joint line. The hypothesis was that a femoral or bifocal deformity is observed in a relevant number of patients with varus malalignment and that a femoral or double-level osteotomy would be necessary in several patients to avoid an oblique joint line.

## Materials and methods

All digital full-leg standing radiographs performed at the authors institution between 2017 and 2019 were reviewed for potential inclusion. For the purpose of this study, only subjects with significant varus alignment, defined as a mechanical tibiofemoral varus angle (mFTA) of ≥ 3° [39, 48], were included. Further inclusion criteria were: Male and female patients, age 18–60 years, and osteoarthritis Grad 0–IV according to Kellgren and Lawrence [27]. Exclusion criteria were: Skeletal immaturity with open growth plates, posttraumatic deformities, previous surgery affecting limb alignment, previous hip, knee, or ankle replacement, and malrotated radiographs with a decentralized patella.

Computer-based deformity analysis and osteotomy simulation was performed using a commercially available planning software (mediCAD^®^, Hectec GmbH, Germany). This software allows for precise analysis of the alignment as well as simulation of single and multiple osteotomies with a high intra- and interrater reliability [18, 44]. All measurements were done by a single observer. Intra- and interrater reliability testing was conducted on 20 randomly chosen and blinded subjects after an interval of 3 weeks.

### Deformity analysis

All digital radiographs were imported to the mediCAD^®^ program and calibrated. Necessary landmarks were marked, including the center of the femoral head, the apex of the greater trochanter, femoral and tibial knee base, medial and lateral border of the femoral condyles and tibial plateau, medial and lateral border of the talus, and the joint line of the talus. Based on these landmarks, all relevant parameters are calculated automatically by the software. For the purpose of this study, the following parameters were recorded: mechanical femorotibial angle (mFTA), position of the weight bearing line (WBL ratio; expressed as % of the medial-to-lateral width of the tibial plateau), mechanical medial proximal tibia angle (mMPTA), mechanical lateral distal femur angle (mLDFA), and joint line convergence angle (JLCA). Based on the amount of varus malalignment, patients were categorized as “mild” (3°–5° varus), “moderate” (6°–8° varus), or “severe” (≥ 9° varus).

Next, the malalignment test as described by Paley et al. [39] was performed to determine the location of the deformity, with normal values for mMPTA and mLDFA of 85°–90° [40]. Based on the deformity location, patients were assorted to one of the following 4 groups:

- Tibial deformity: mMPTA < 85°, mLDFA normal
- Femoral deformity: mLDFA > 90°, mMPTA normal
- Tibial + femoral deformity: mMPTA < 85° and mLDFA > 90°
- No bony deformity: mMPTA and mLDFA normal

Given a considerable number of patients without a true bony deformity as defined above, patients were further analyzed regarding their potential for bony correction based on deviations from the upper or lower limit of the mMPTA and mLDFA, respectively:

- Tibial potential: mMPTA < 90°
- Femoral potential: mLDFA > 85°
- Tibial and femoral potential: mMPTA < 90° and mLDFA > 85°
- No potential: mMPTA ≥ 90° and mLDFA ≤ 85°

### Osteotomy simulation

**Fig. 1:**
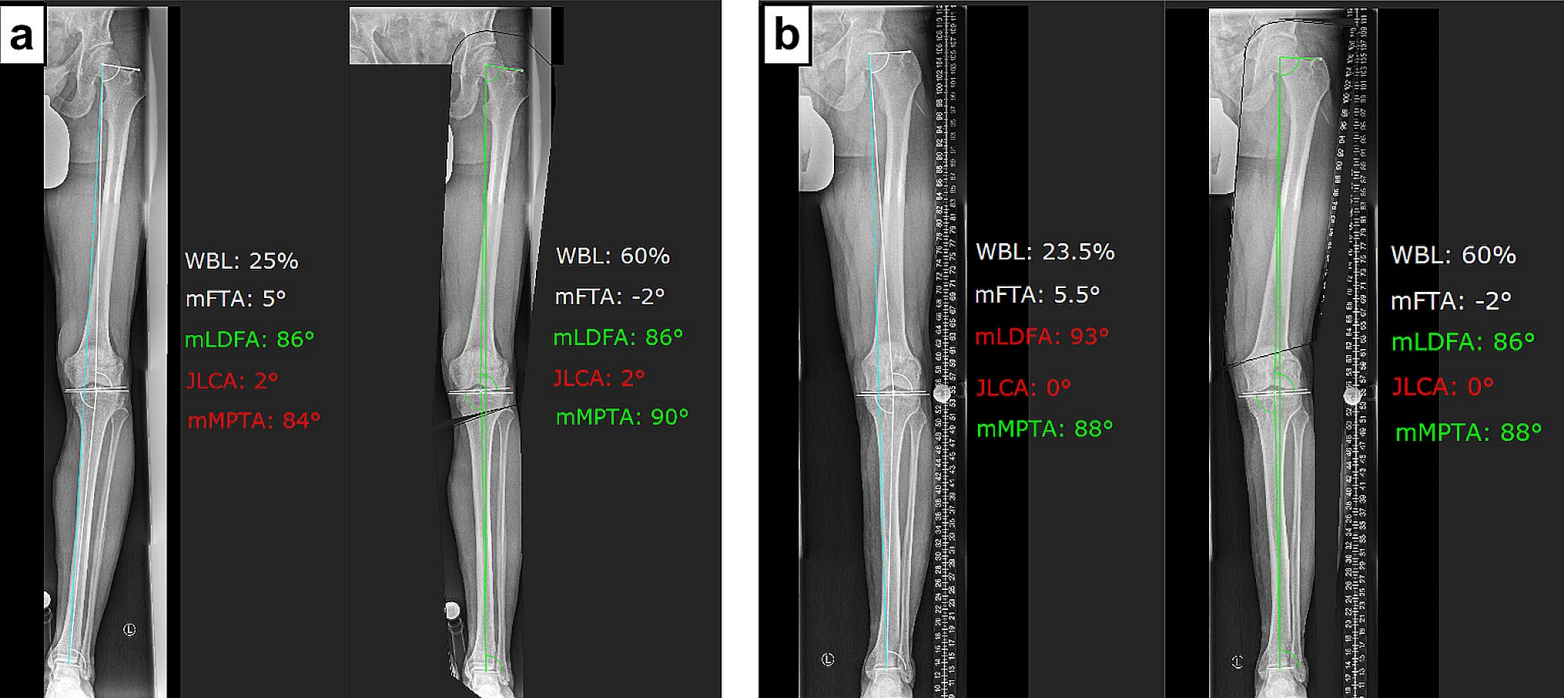
Illustrative case examples 1 and 2. **a** Case 1: deformity analysis revealed a tibial-based varus deformity of 5° with a normal mLDFA and a pathologic mMPTA. This deformity can be corrected via medial open-wedge HTO to the desired alignment of 2° of valgus without exceeding the upper limit of the mMPTA. **b** Case 2: deformity analysis revealed a femoral-based varus deformity of 5.5° with a normal mMPTA and a pathologic mLDFA. This deformity can be corrected via lateral closed-wedge DFO to the desired alignment of 2° of valgus without exceeding the lower limit of the mLDFA

**Fig. 2:**
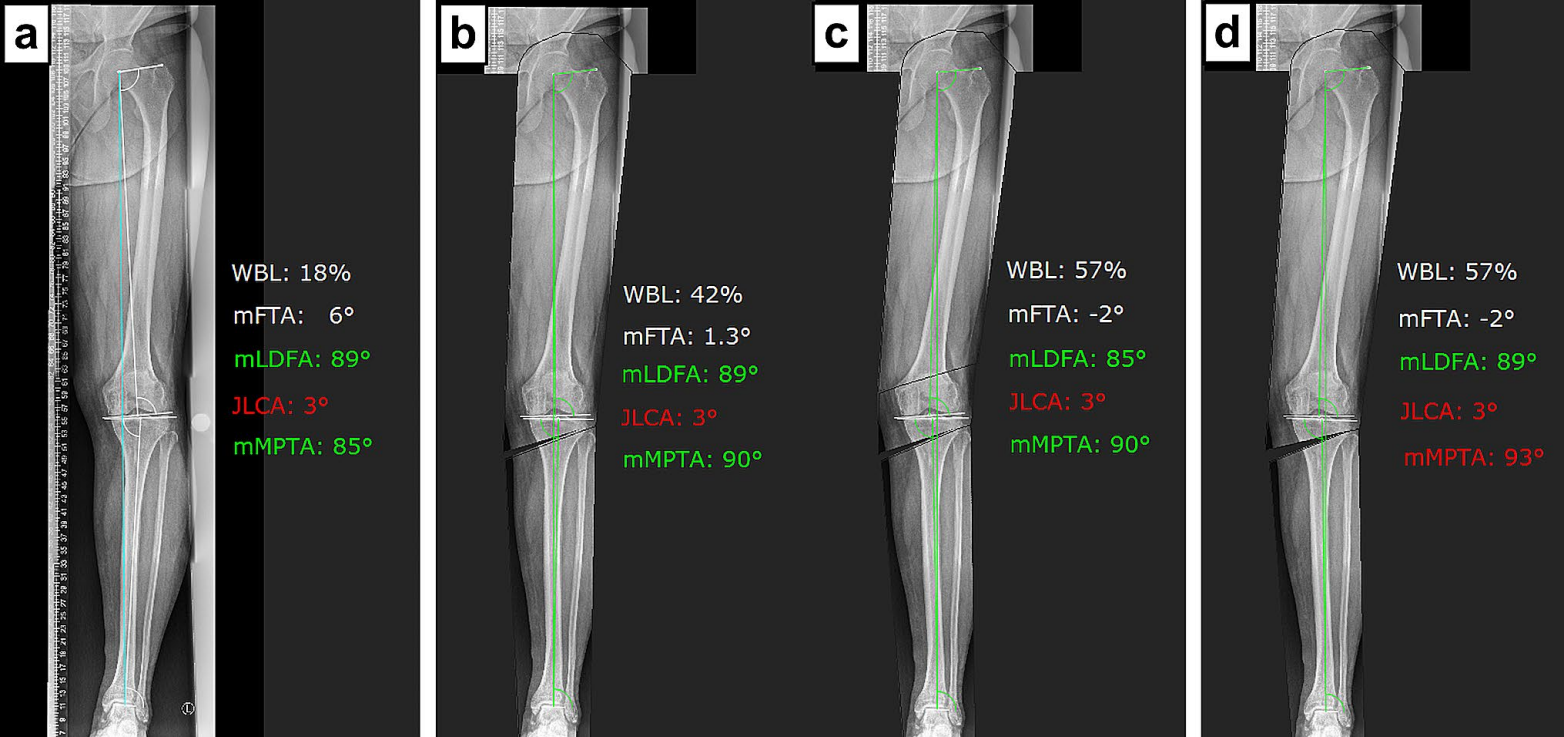
Illustrative case example 3. **a** Deformity analysis revealed a varus deformity of 6° without a true bone deformity based on the malalignment test [39]. However, potential for bony correction exist in both, the proximal tibia and distal femur, with the greater potential being located at the proximal tibia. **b**, **c** First osteotomy simulation tolerating mLDFA ≥ 85° and mMPTA ≤ 90° (anatomic correction): by simulating HTO alone, 1.3° of varus alignment remains with the mMPTA set at 90°. By simulating a double-level osteotomy, the deformity can be corrected to the desired alignment of 2° of valgus without exceeding the upper and lower limit of the mMPTA and mLDFA, respectively. **d** Second osteotomy simulation tolerating mLDFA ≥ 85° and mMPTA ≤ 95° (overcorrection): the deformity can be corrected via HTO to the desired alignment of 2° of valgus without exceeding the upper limit of the mMPTA of 95°

**Fig. 3:**
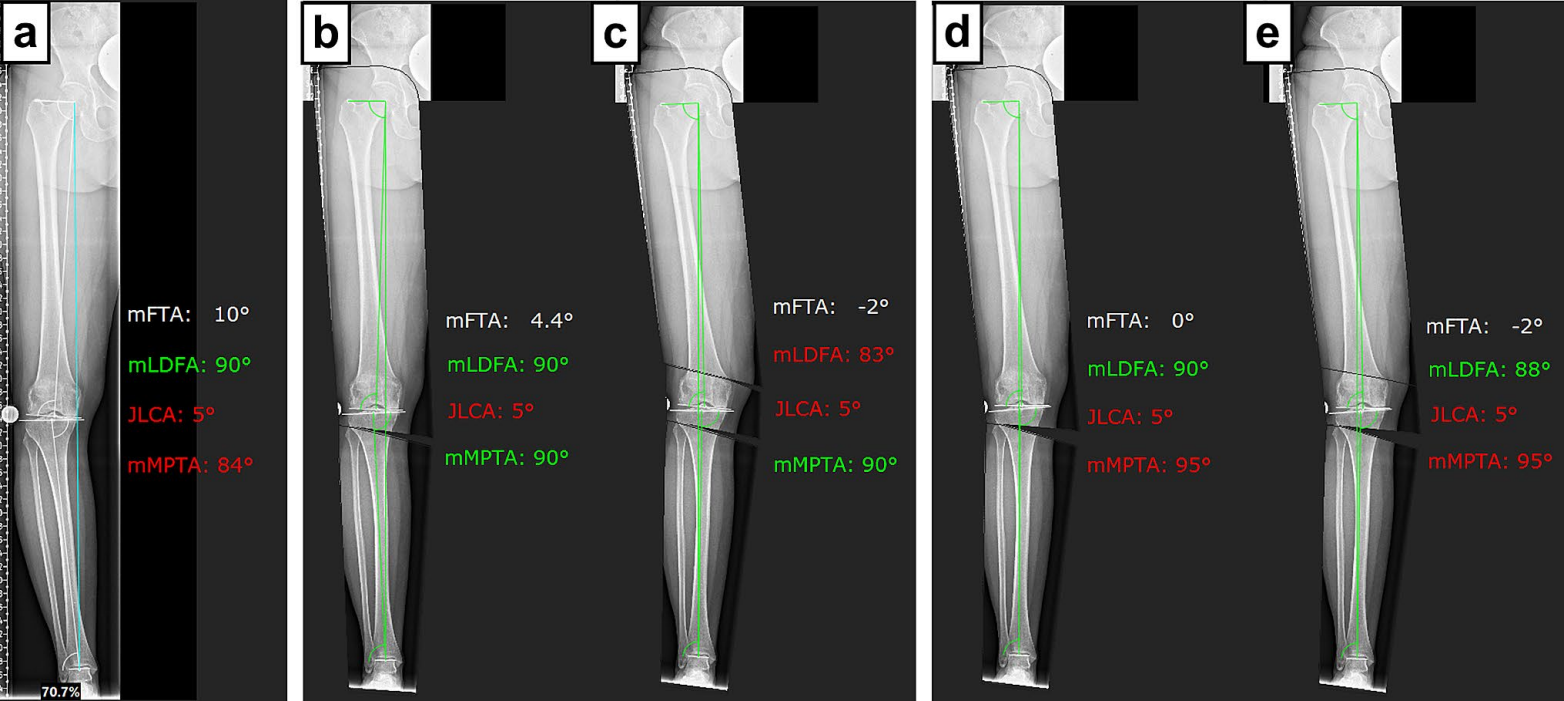
Illustrative case example 4. **a** Deformity analysis revealed a tibial-based varus deformity of 10° with a high-normal mLDFA and a pathologic mMPTA. **b**, **c** First osteotomy simulation tolerating mLDFA ≥ 85° and mMPTA ≤ 90° (anatomic correction): by simulating HTO alone, 4.4° of varus alignment remains with the mMPTA set at 90°. By simulating a double-level osteotomy to the desired alignment of 2° of valgus, the lower limit of the mLDFA is exceeded. This case is, therefore, considered “uncorrectable”. **d**, **e** Second osteotomy simulation tolerating mLDFA ≥ 85° and mMPTA ≤ 95° (overcorrection): by simulating HTO alone, neutral alignment remains with the mMPTA set at 95°. By simulating a double-level osteotomy, the deformity can be corrected to the desired alignment of 2° of valgus without exceeding the upper and lower limit of the mMPTA and mLDFA, respectively

Simulation of the osteotomy always started at the site of the greatest deformity as revealed in the malalignment test: In case of a tibial-based deformity, a medial open-wedge HTO was simulated and in case of a femoral-based deformity a lateral closed-wedge DFO was simulated. If no true bony deformity was present, the primary osteotomy site was chosen based on the greatest amount of bony correction potential. All legs were corrected to a postoperative mFTA of 2° valgus [50]. To avoid an oblique joint line, limits for the postoperative knee base angles were defined. For the mLDFA, the postoperative lower limit was set at 85° for all simulations [36]. With regard to the mMPTA, two different simulations were performed: one simulation with a postoperative upper limit of 90° (anatomic correction) and another simulation with a postoperative upper limit of 95° (overcorrection). If the simulation of a single osteotomy (HTO or DFO) led to deviation from these limits at the intended mFTA of 2° valgus, a double-level osteotomy was simulated. In this case, the first osteotomy was simulated at the site of the greatest deformity and the corresponding knee base angle was corrected to its upper or lower limit, respectively. The second osteotomy was simulated at the opposite site until the intended alignment of 2° of mechanical valgus was achieved. Based on these simulations, the ideal osteotomy level was classified as “tibial”, “femoral”, or “double-level”. If even a double-level osteotomy resulted in deviations from the defined limits for knee base angles, the leg was categorized as “uncorrectable”. Illustrative case examples are shown in Figs. 1, 2, 3.

### Statistical analysis

Statistical analysis was performed using SPSS software version 25.0 (IBM-SPSS, New York, USA). Continuous variables were calculated as mean ± standard deviation and categorical variables as count and percentages.

Intraclass correlation coefficients (ICCs) were calculated to determine the intra- and interobserver reproducibility of the obtained measurements.

Normal distribution of all data was evaluated with the Kolmogorov–Smirnov test.

Distribution of deformity location and ideal osteotomy level with regard to the amount of varus malalignment (“mild” vs. “moderate” vs. “severe”) and osteotomy simulation (anatomic vs. overcorrection) was compared with the Qui-square test followed by post hoc tests with Bonferroni correction of the *p* value. JLCA between knees with “mild”, “moderate”, or “severe” varus was compared with the Kruskal–Wallis test followed by post hoc analysis with Bonferroni correction.

This study was approved by the Ethics Committee of the Technical University of Munich.

## Results

A total of 303 full-leg standing radiographs could be included. Patient demographics are provided in Table 1.

**Table 1:** Patient demographics of the total study group

Number of patients	303
Sex	
Female	24% (72)
Male	76% (231)
Age (years)	44 ± 11 (18–60)
Laterality	
Left	55% (165)
Right	46% (138)
Osteoarthritis according to Kellgren and Lawrence	
No OA	11% (34)
Grade I	34% (104)
Grade II	29% (88)
Grade III	18% (53)
Grade IV	8% (24)
Varus deformity	
Mild (3°–5°)	59% (178)
Moderate (6°–8°)	32% (98)
Severe (≥ 9°)	9% (27)

Continuous variables are shown as mean ± standard deviation and (range), categorical variables are shown as percentages per group and (number of patients)

### Deformity analysis

**Fig. 4:**
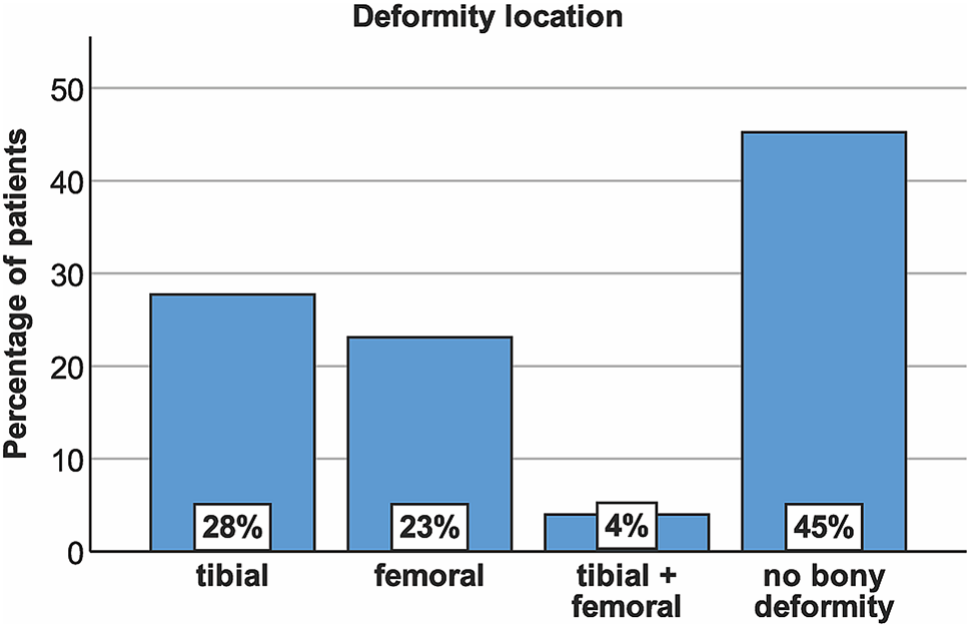
Deformity location based on the malalignment test [39] with normal values for mMPTA and mLDFA of 85°–90° [40]. Tibial deformity: mMPTA < 85°, mLDFA normal; femoral deformity: mLDFA > 90°, mMPTA normal; tibial + femoral deformity: mMPTA < 85° + mLDFA > 90°; no bony deformity: mMPTA + mLDFA normal

Measurements of the deformity analysis and corresponding ICC values are shown in Table 2. For the total study population, the malalignment test revealed a tibial deformity in 28%, a femoral deformity in 23%, a combined tibial and femoral deformity in 4%, and no bony deformity in 45% (Fig. 4). Potential for bony correction was observed in all patients, with almost all patients demonstrating potential for correction at the tibial and femoral site (94%). The greatest potential for correction was observed at the tibial site in 56%.

**Table 2:** Measurements of the deformity analysis and corresponding intraclass correlation coefficients

	Mean ± SD	Median	Range	Intrarater ICC	Interrater ICC
mFTA	6° ± 11°	5°	3°–15°	0.997	0.996
WBL ratio	23 ± 8%	24%	1–39%	0.994	0.992
JLCA	2° ± 2°	2°	0°–8°	0.903	0.940
mMPTA	86° ± 2°	86°	78°–93°	0.991	0.984
mLDFA	89° ± 2°	89°	83°–95°	0.981	0.965

*SD* standard deviation, *ICC* intraclass correlation coefficient, *mFTA* mechanical femorotibial angle, *WBL* weight bearing line, *JLCA* joint line convergence angle, *mMPTA* mechanical medial proximal tibial angle, *mLDFA* mechanical lateral distal femoral angle

Deformity location with regard to the amount of varus malalignment is summarized in Table 3. No significant difference was observed between the three groups (“mild” vs. “moderate” vs. “severe”) for the prevalence of a tibial deformity. Compared to “mild” varus, a femoral deformity was significantly more prevalent in “moderate” and “severe” varus (*p* = 0.016 and *p* = 0.008), and a combined femoral + tibial deformity was significantly more prevalent in “severe” varus as compared to “mild” and “moderate” varus (*p* < 0.001 and *p* = 0.011). No bony deformity was significantly more prevalent in “mild” varus as compared to “moderate” and “severe” varus (*p* < 0.001), and significantly more prevalent in “moderate” varus as compared to “severe” varus (*p* = 0.034). Comparison of JLCA between the three groups revealed a significantly higher JLCA in “moderate” and “severe” varus as compared to “mild” varus (*p* = 0.002 and *p* < 0.001).

**Table 3:** Deformity location based on the malalignment test [39] with regard to the amount of varus malalignment

	Varus malalignment (mFTA)		
	Mild (3°–5°)	Moderate (6°–8°)	Severe (≥ 9°)
Deformity location			
Tibial (mMPTA < 85°, mLDFA normal)	23%	35%	33%
Femoral (mLDFA > 90°, mMPTA normal)	16%	31%^a^	41%^a^
Tibial + femoral (mMPTA < 85° + mLDFA > 90°)	2%	3%	19%^b^
No deformity (mMPTA + mLDFA normal)	58%^c^	32%^d^	7%
JLCA	1.8° ± 1.3° (0.1°–6.4°)	2.4° ± 1.6^e^ (0.1°–7.7°)	3.3° ± 1.8^e^ (0.2°–6.8°)

Normal values for mMPTA and mLDFA were 85°–90° [39, 40]

Values are shown as percentages per group or mean ± standard deviation and range

*mFTA* mechanical femorotibial angle, *mMPTA* mechanical medial proximal tibial angle, *mLDFA* mechanical lateral distal femoral angle, *JLCA* joint line convergence angle

^a^Significant difference between 3°–5° and 6°–8° mFTA (*p* = 0.016) and between 3°–5° and ≥ 9° mFTA (*p* = 0.008) (Qui-square test followed by post hoc tests with Bonferroni correction)

^b^Significant difference compared to 3°–5° and 6°–8° mFTA (*p* < 0.001 and *p* = 0.011) (Qui-square test followed by post hoc tests with Bonferroni correction)

^c^Significant difference compared to 6°–8° and ≥ 9° mFTA (*p* < 0.001) (Qui-square test followed by post hoc tests with Bonferroni correction)

^d^Significant difference compared to ≥ 9° mFTA (*p* = 0.034) (Qui-square test followed by post hoc tests with Bonferroni correction)

^e^Significant difference between 3°–5° and 6°–8° mFTA (*p* = 0.002) and between 3°–5° and ≥ 9° mFTA (*p* < 0.001) (Kruskal–Wallis test followed by post hoc analysis with Bonferroni correction)

### Osteotomy simulation

**Fig. 5:**
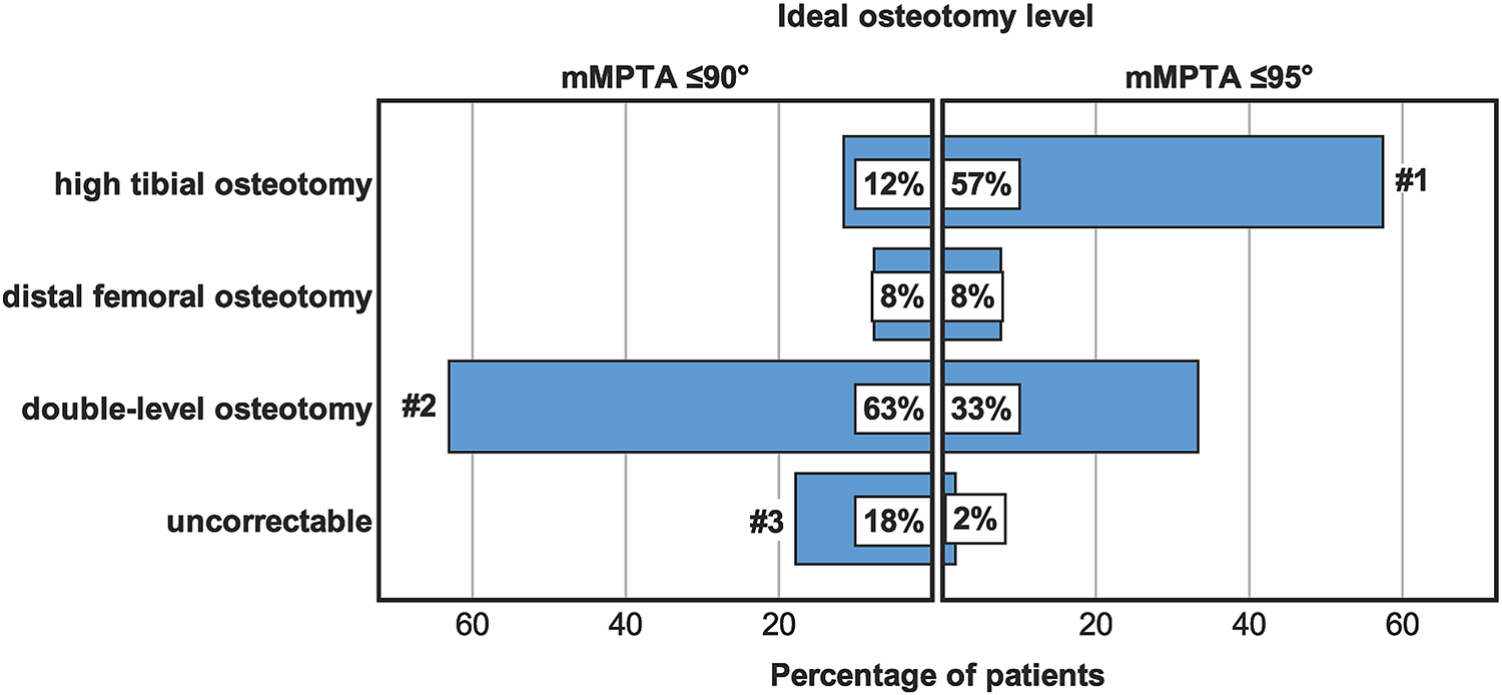
Ideal osteotomy level tolerating a mechanical medial proximal tibial angle (mMPTA) of ≤ 90° (anatomic correction) or ≤ 95° (overcorrection). #1 significant difference compared to mMPTA ≤ 90° (*p* < 0.001); #2 significant difference compared to mMPTA ≤ 95° (*p* < 0.001); #3 significant difference compared to mMPTA ≤ 95° (*p* < 0.001) (Qui-square test followed by post hoc tests with Bonferroni correction)

Distribution of the ideal osteotomy level for both simulations (mMPTA ≤ 90° and mMPTA ≤ 95°) is shown in Fig. 5. An isolated HTO was appropriate in 12% for an anatomic correction, whereas a double-level osteotomy was necessary in 63%. If overcorrection was tolerated, the number of HTOs significantly increased to 57% (*p* < 0.001), whereas the number of double-level osteotomies significantly decreased to 33% (*p* < 0.001). An isolated DFO was considered ideal in 8% for both simulations.

Distribution of the ideal osteotomy level with regard to the amount of varus malalignment is summarized in Tables 4 and 5. By simulating an anatomic correction, HTO was significantly more often appropriate in knees with “mild” varus, as compared to knees with “moderate” or “severe” varus (*p* < 0.001). Knees with “severe” varus were significantly more frequently considered uncorrectable, as compared to knees with “mild” or “moderate” varus (*p* < 0.001 and *p* = 0.034). By simulating overcorrection of the mMPTA, HTO was significantly more often appropriate in knees with “mild” and “moderate” varus, as compared to knees with “severe” varus (*p* < 0.001 and *p* = 0.005). Compared to “mild” and “moderate” varus, a double-level osteotomy was significantly more often necessary in knees with “severe” varus (*p* < 0.001 and *p* = 0.001).

**Table 4:** Ideal osteotomy level tolerating a mechanical lateral distal femoral angle (mLDFA) of ≥ 85° and a mechanical medial proximal tibial angle (mMPTA) of ≤ 90° (anatomic correction) with regard to the amount of varus malalignment

Ideal osteotomy level	Varus malalignment (mFTA)		
	Mild (3°–5°) (%)	Moderate (6°–8°) (%)	Severe (≥ 9°) (%)
Tibial	18^a^	3	0
Femoral	11	4	0
Double-level	59	72	56
Uncorrectable	12	20	44^b^

Values are shown as percentages per group

*mFTA* mechanical femorotibial angle

^a^Significant difference compared to 6°–8° and ≥ 9° mFTA (*p* < 0.001) (Qui-square test followed by post hoc tests with Bonferroni correction)

^b^Significant difference compared to 3°–5° and 6°–8° (*p* < 0.001 and *p* = 0.034) (Qui-square test followed by post hoc tests with Bonferroni correction)

**Table 5:** Ideal osteotomy level tolerating a mechanical lateral distal femoral angle (mLDFA) of ≥ 85° and a mechanical medial proximal tibial angle (mMPTA) of ≤ 95° (overcorrection) with regard to the amount of varus malalignment

Ideal osteotomy level	Varus malalignment (mFTA)		
	Mild (3°–5°) (%)	Moderate (6°–8°) (%)	Severe (≥ 9°) (%)
Tibial	64^a^	56^a^	22
Femoral	11	4	0
Double-level	24	38	78^b^
Uncorrectable	2	2	0

Values are shown as percentages per group

*mFTA* mechanical femorotibial angle

^a^Significant difference between 3°–5° and 6°–8° mFTA (*p* < 0.001) and between 3°–5° and ≥ 9° mFTA (*p* = 0.005) (Qui-square test followed by post hoc tests with Bonferroni correction)

^b^Significant difference compared to 3°–5° and 6°–8° (*p* < 0.001 and *p* = 0.001) (Qui-square test followed by post hoc tests with Bonferroni correction)

## Discussion

The most important findings of the present study were that only 28% of patients with varus malalignment ≥ 3° had a tibial deformity based on the malalignment test described by Paley. Most patients (45%) had no bony deformity, and another 23% had a femoral deformity. If anatomic correction (mMPTA ≤ 90°, mLDFA ≥ 85°) was intended, only 12% of patients could be corrected via isolated HTO, whereas 63% of patients required a double-level osteotomy. If slight overcorrection at the tibial site was accepted (mMPTA ≤ 95°), 57% of patients could be corrected via isolated HTO, whereas 33% of patients still required a double-level osteotomy, and 8% were best corrected via isolated DFO.

Valgus-producing HTO has been used for several decades as a surgical treatment for medial compartment OA associated with varus malalignment [10, 11]. With continuous improvements in surgical technique and the introduction of angle-stable implants, indications for corrective osteotomies have been extend [15, 30], and HTO is nowadays regularly performed as a concomitant procedure in patients undergoing cartilage repair procedures [6, 32, 33], meniscal transplantation [52], or ligament reconstruction [49]. Most clinical outcome data are available for the treatment of medial compartment OA, and valgus HTO can be considered an evidence-based procedure [9]. Several negative predictive factors for worse outcome and failure have been reported including increased age, high BMI, advanced OA, and under- or overcorrection [8, 14, 24]. Another important factor to consider is postoperative joint line obliquity [4, 5, 11]. This phenomenon can occur if HTO is performed in a normally aligned tibia, or if a severe varus deformity is corrected via HTO alone [36, 37]. Beside difficulties in converting to total knee arthroplasty [20], an oblique joint line and particularly excessive overcorrection of the mMPTA has been associated with worse clinical outcome and higher failure rates after HTO [1, 4, 11, 47]. However, no consensus exists to what extent overcorrection of the mMPTA is acceptable [17]. Using a 3D finite element model analysis, Nakayama et al. [37] could demonstrate that HTO induced excessive shear stress in the medial compartment if joint line obliquity was 5° or more. The authors, therefore, proposed a double-level osteotomy in varus knees with a preoperatively anticipated mMPTA > 95° [37]. This proposal is affirmed by two clinical studies: Akamatsu et al. [1] have shown that patients with a postoperative mMPTA > 95° had worse knee function at 2 years after medial open-wedge HTO. Furthermore, Schuster et al. [47] found inferior long-term functional outcome in patients with a postoperative mMPTA > 95° at 10 years after medial open-wedge HTO. On the other hand, Goshima et al. [17] reported that an overcorrected mMPTA of > 95° did not affect the clinical outcome after a minimum follow-up of 2 years. It is important to note, that a mMPTA of 95° does not necessarily imply joint line obliquity of 5°. More specifically, it has been demonstrated that changes of joint line obliquity are smaller than changes of the mMPTA because of compensatory changes in the hip and ankle joints [17, 29, 38]. Whereas a certain amount of overcorrection seems to be acceptable, further studies are necessary to better understand the interaction between mMPTA and joint line obliquity and their impact on outcomes after realignment osteotomies.

To avoid excessive overcorrection of the mMPTA and joint line obliquity, detailed deformity analysis and precise planning of the osteotomy are paramount [5, 43]. Several methods have been proposed, with most of them being based on mFTA measurement or the Mikulicz line [12, 31]. However, based on the findings of the present study, deformity analysis should include measurement of the knee base angles (mMPTA and mLDFA) to determine the origin of the varus deformity. This study found that only 28% of patients with varus malalignment had a tibial-based deformity. Most patients (45%) did not show a bony deformity based on the malalignment test of Paley. In those patients, varus malalignment is either the result of intraarticular wear and/or lateral ligament laxity, or the result of small deviations of the knee base angles in both the femur and tibia. Especially patients with varus due to intraarticular wear are no good candidates for corrective osteotomies and should best be treated with unicompartmental knee arthroplasty. On the other hand, some patients may be good candidates for corrective osteotomies, despite the lack of a true bony deformity. However, a high proportion of these patients will require a double-level osteotomy to avoid an oblique joint line. The same is true in patients with severe varus malalignment despite a bony deformity [36, 45]. In the present study, a double-level osteotomy was considered ideal in 63% if anatomic correction was intended and in 33% if overcorrection of the mMPTA to 95° was accepted. Furthermore, a double-level osteotomy was significantly more often necessary in knees with sever varus malalignment. The concept of double-level osteotomy has been introduced to restore physiologic alignment and knee base angles [5, 36, 41, 45]. Whereas older studies have observed poor results and unacceptable high complication rates [46], more recent studies have shown that double-level osteotomy is a safe procedure which enables accurate and consistent deformity correction with good clinical results and low failure rates [5, 36, 41, 45]. Nevertheless, double-level osteotomies are technically demanding and more invasive compared to isolated HTO. Furthermore, comparative studies between HTO and double-level osteotomies are lacking. Further studies are, therefore, necessary to prove the advantage of double-level osteotomies.

According to the results of the present study, 8% of varus deformities should be corrected at the femur via isolated DFO. However, only limited data exists about the clinical efficiency of DFO in varus knees. Van der Woude et al. [51] analyzed the results of closed-wedge valgus DFO in 15 patients with a mean age of 45 years. After a mean follow-up of 40 months, the authors reported clinical improvement and accurate correction [51]. However, from a biomechanical point of view, it must be noted, that DFO decreases tibiofemoral contact pressure more effectively in extension compared with increasing angles of knee flexion [53]. Based on knowledge of total knee arthroplasty, HTO should decrease tibiofemoral contact pressure throughout flexion angles [53]. Therefore, it remains unknown whether DFO is as effective as HTO. To date, only one study has reported results after both, DFO and HTO to correct varus malalignment. Fürmetz et al. [16] prospectively evaluated 25 consecutive patients undergoing realignment osteotomy of a varus deformity. HTO was performed in 17 patients and DFO in 11 patients. After a mean follow-up of 47 months, improvement in all clinical scores was observed, without differences between the two techniques [16]. However, the small patient cohort and relatively short follow-up period limit the conclusion of this study. Since most of the weightbearing occurs during the stance phase of gait in full extension [53], the authors believe that valgus DFO may be as effective as HTO. However, further comparative studies are necessary to proof this assumption.

This study has several limitations which must be considered. First, patients were included based on radiographs without taken clinical symptoms into consideration. Therefore, our analysis must be regarded as a cross section of varus alignment in general. It remains unknown whether the geometry of a varus knee differs with regard to the specific pathologies. However, to obtain a representative cohort for corrective osteotomies, patients < 18 and > 60 years were excluded. Second, alignment was measured on radiographs, which only represents a 2D projection of a three-dimensional structure. Utilization of 3D-reconstructed CT images may, therefore, be more accurate, since bony landmarks can be determined more precisely [22]. Nevertheless, standing full-leg radiographs are the current standard for the assessment of coronal limb in the clinical practice [13]. Third, varus malalignment was defined as a mechanical tibiofemoral varus angle of ≥ 3° whereas other authors may consider ≥ 5° as an indication for realignment osteotomies. However, indications have evolved during the last decade and realignment osteotomies are nowadays considered in patients with < 5° [35], especially when performed as a concomitant procedure with cartilage repair procedures [6, 7, 33]. The authors, therefore, believe that patients with only mild varus deformity are also important to consider. Fourth, all osteotomies were simulated to a postoperative alignment of 2° of mechanical valgus. However, there is no consensus about the “ideal” postoperative alignment and an individualized approach based on the indication for osteotomy has been proposed [15]. Nevertheless, a target of 2°–3° of mechanical valgus is common in the literature [50]. Fifth, the method used for osteotomy simulation does only take static alignment into consideration. Changes of JLCA and compensatory changes of the hip and ankle joints are not included. Nevertheless, these parameters may influence postoperative knee joint obliquity, as discussed above. Furthermore, in knees with a large preoperative JLCA, overcorrection may have occurred with our simulation technique and less bony correction would have been required to obtain the target postoperative alignment [28]. However, it remains unclear to what extent preoperative JLCA changes after valgus osteotomy. Some studies have shown that the absolute changes after HTO are small, with differences in mean JLCA ≤ 1° [2, 28]. Given these small changes in JLCA and the fact that 82% of our patients had a JLCA of only 0°–3°, we do not belief that differences in JLCA introduced a major bias. Nevertheless, further developments in computer-based osteotomy planning should take dynamic variables into consideration.

Despite these limitations, this study underlines the importance of meticulous deformity analysis and precise osteotomy planning. Varus malalignment should not uniformly be considered a tibial-based deformity and hence be corrected via HTO. Instead, an individualized approach is recommended. A computer-based planning software which is able to simulate postoperative values for mMPTA and mLDFA is of great advantage to define the ideal osteotomy level [43]. In addition, computer-based planning software allows simulation of double-level osteotomies, which is difficult with conventional planning methods. If no dedicated planning software is available, conventional methods can be utilized such as the Miniaci method [31]. However, before osteotomy planning, mFTA and knee base angles must be measured and it must be estimated if the intended correction can be achieved via isolated HTO. Furthermore, we recommend to measure the resulting mMPTA to control for excessive overcorrection.

## Conclusion

Less than one-third of patients with varus malalignment ≥ 3° have a tibial deformity with mMPTA < 85°. If anatomic correction (mMPTA ≤ 90°, mLDFA ≥ 85°) is intended, only 12% of patients can be corrected via isolated HTO, whereas 63% of patients would require a double-level osteotomy. If slight overcorrection at the tibial side is accepted (mMPTA ≤ 95°), 57% of patients can be corrected via isolated HTO, whereas 33% of patients would require a double-level osteotomy, and 8% should be corrected via isolated DFO.
